# An Innovative Technique of Testicular Preservation in Fournier’s Gangrene: Surgical Details and Illustration

**DOI:** 10.7759/cureus.27581

**Published:** 2022-08-01

**Authors:** Ashok Puranik, Suruthi Baskaran, Ravi R Kumar

**Affiliations:** 1 General Surgery, All India Institute of Medical Sciences, Jodhpur, Jodhpur, IND

**Keywords:** fournier gangrene, surgical technique guide, urogenital pathology, shameful testes, scrotal surgery

## Abstract

Fournier’s gangrene, which is a necrotizing fasciitis of the perineal region, requires prompt control of infection with emergent surgical debridement. The shameful exposure of gonads, which occurs following debridement, can cause both physiological and psychological impairment to the patient. These can be avoided by the use of this novel technique for testicular preservation.

Following debridement of necrotic scrotal skin, this technique involves creation of inguinal pouch by blunt dissection and placement of the testes in the pouch created. Once healthy granulation tissue is achieved in the scrotal wound, closure of the scrotum is performed after bringing down the testes. The advantages of this technique include development of a relatively physiological position to preserve the testes before definitive reconstruction of the scrotum and the easy reproducibility of the technique.

A holistic approach to management of Fournier’s gangrene should include resuscitation, administration of antibiotics, debridement, and scrotal reconstruction. However, the psychological impact of shameful exposure of the gonads must also be borne in mind during the management. Our technique represents one of the ways to reduce the stigma and discomfort associated with shameful exposure of the testes.

## Introduction

Fournier’s gangrene, which is a necrotizing fasciitis of the perineal region, has always posed a unique and stressful problem to both the patient and the surgeon. This spreading gangrene of the scrotal tissues can involve the genital, perianal, and surrounding tissues of the thigh or abdominal wall, requiring rapid resuscitation, debridement, and administration of appropriate antibiotics [1]. The resultant defect of the scrotal wall often leads to shameful exposure of the testes, which creates a distinctive psychological stress to the patient including aesthetic complications and physiological stress to the testes. This paper describes the surgical details with illustrations of a novel technique of testicular preservation following extensive debridement for Fournier’s gangrene by placing the testes in a surgically created inguinal pouch, thereby preventing exposure of healthy gonads to the environment.

## Technical report

Description of the technique

The management of Fournier’s gangrene essentially starts with adequate resuscitation with intravenous fluids and antibiotics along with emergent surgical debridement. The steps of this innovative technique of inguinal pouch creation for Fournier’s gangrene management include the following.

Step 1: Debridement of Necrotic Scrotal Skin

**Figure 1:**
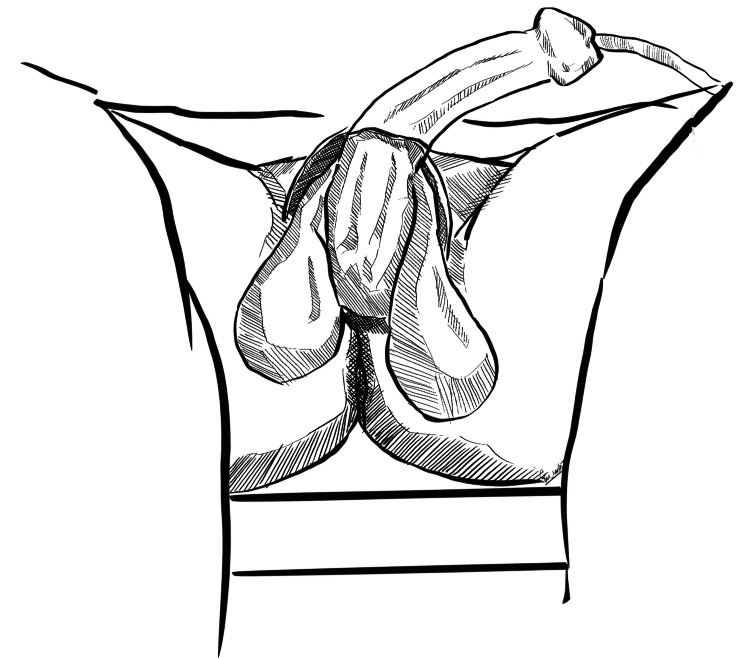
Exposed testes following debridement The testes are shamefully exposed after removal of necrotic scrotal skin. Original image created by Sreeshanth KS

Extensive debridement of necrotic scrotal skin and surrounding tissues is to be performed, ensuring healthy margins with active bleeding (Figure 1). Testes are usually found to be healthy and preserved. After removal of necrotic tissues, thorough wash with saline is performed.

Step 2: Creation of Inguinal Pouch

**Figure 2:**
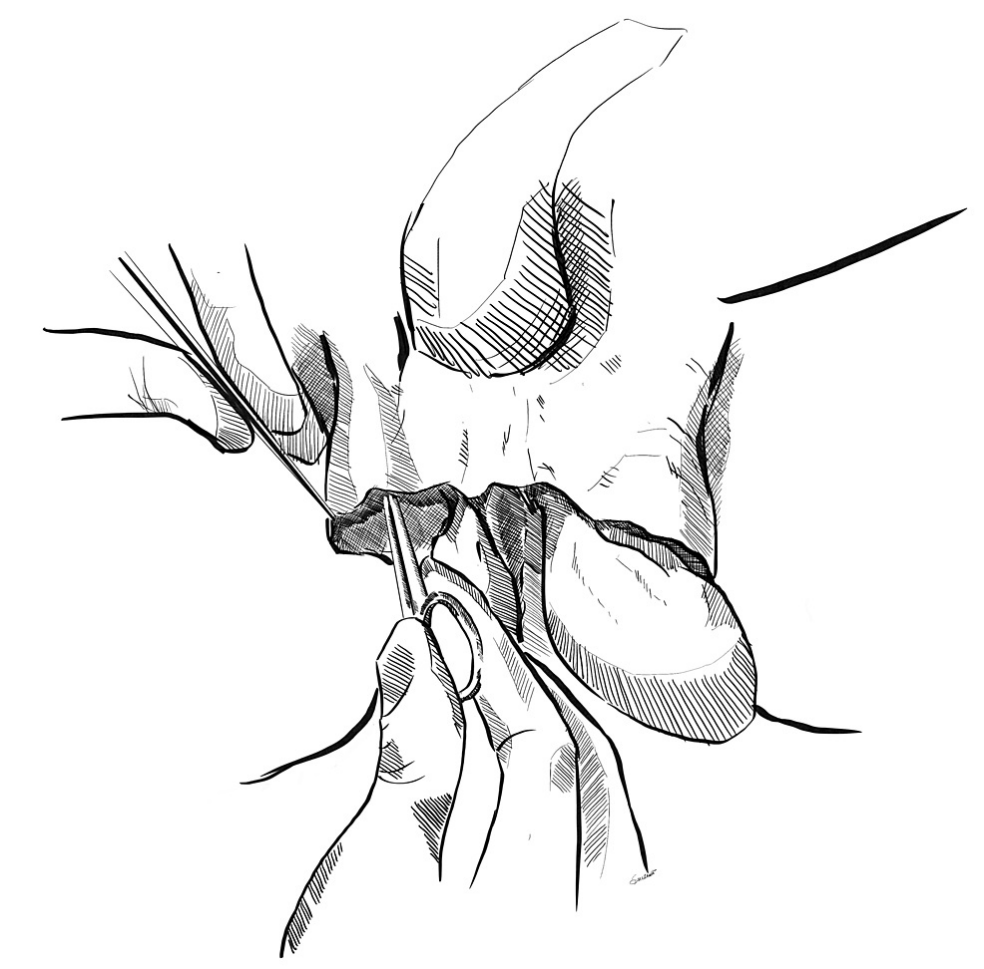
Creation of the right inguinal pouch A gauze piece held with a sponge holder is used to create space via blunt dissection on the right side. Original image created by Sreeshanth KS

**Figure 3:**
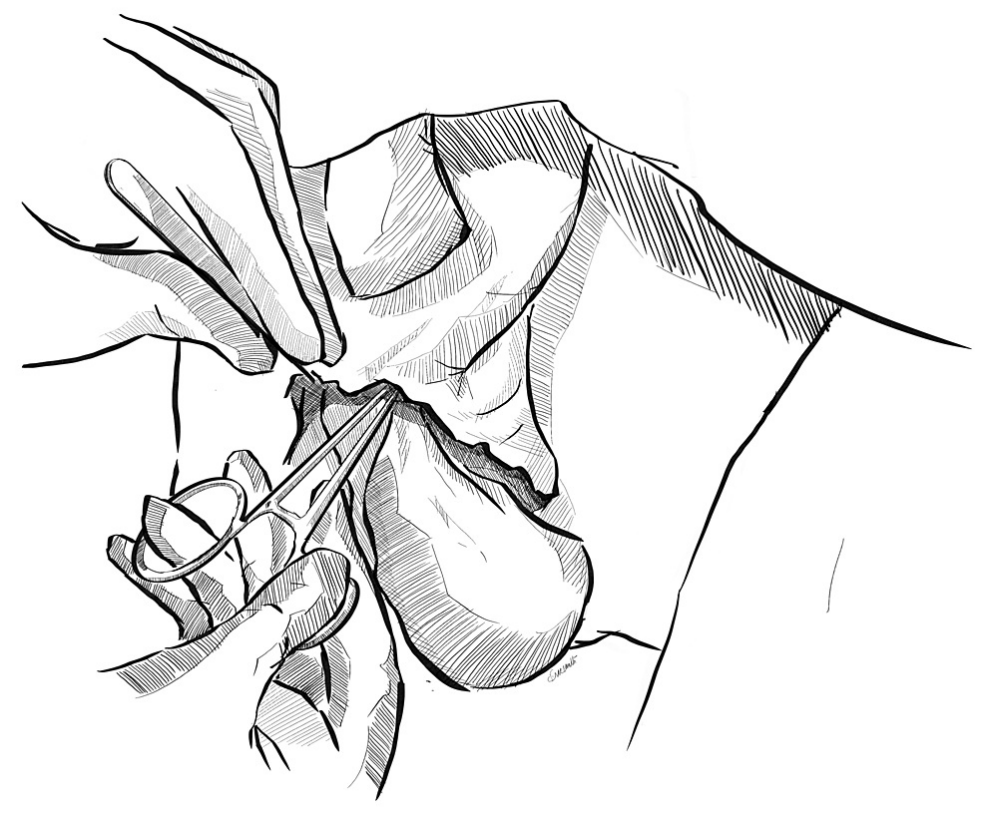
Creation of the left inguinal pouch A gauze piece held with a sponge holder is used to create space via blunt dissection on the left side. Original image created by Sreeshanth KS

After primary debridement and thorough washing, space is created in the inguinal region by blunt dissection. Two retractors are placed at the level of superficial inguinal ring to expose the inguinal canal. Adequate space is created in the bilateral inguinal canal, anterior to the inguinal cord, with blunt finger dissection or with the use of a gauze piece held at the tip of an artery forceps or a sponge holder (Figures 2, 3).

Step 3: Placement of Testes in the Inguinal Pouch

**Figure 4:**
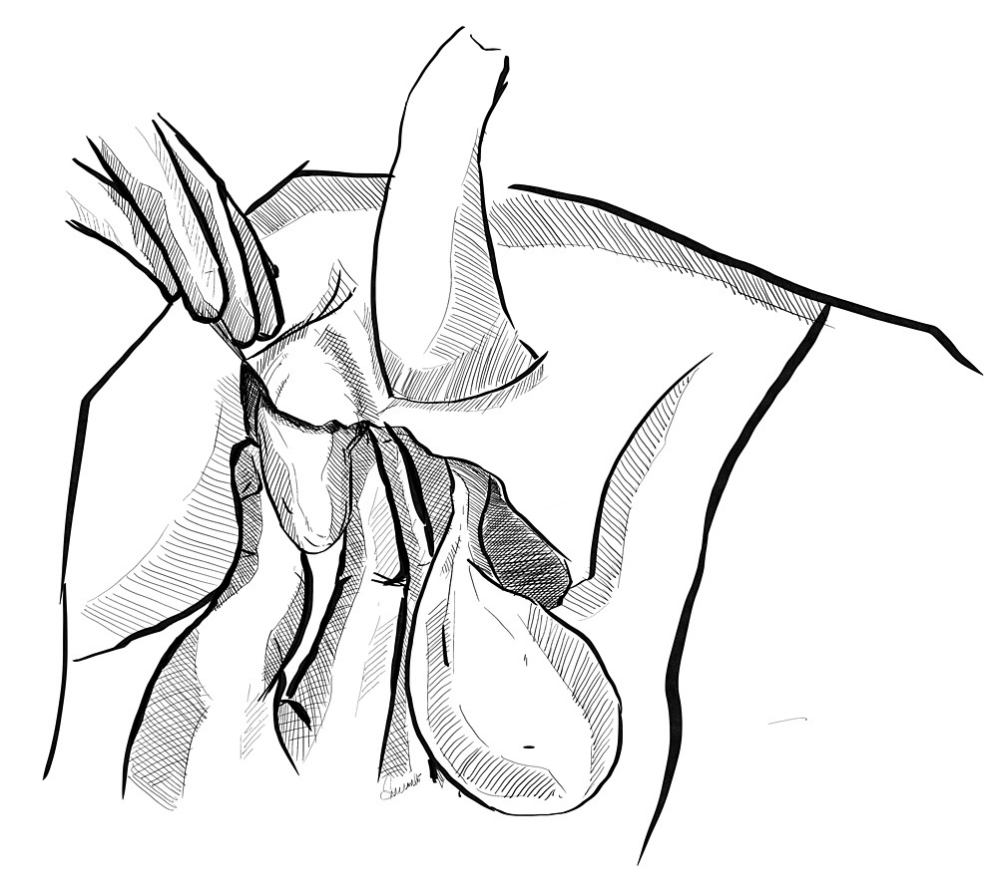
Placement of the testis in the right inguinal pouch The right testis is gently pushed manually into the inguinal pouch created. Original image created by Sreeshanth KS

**Figure 5:**
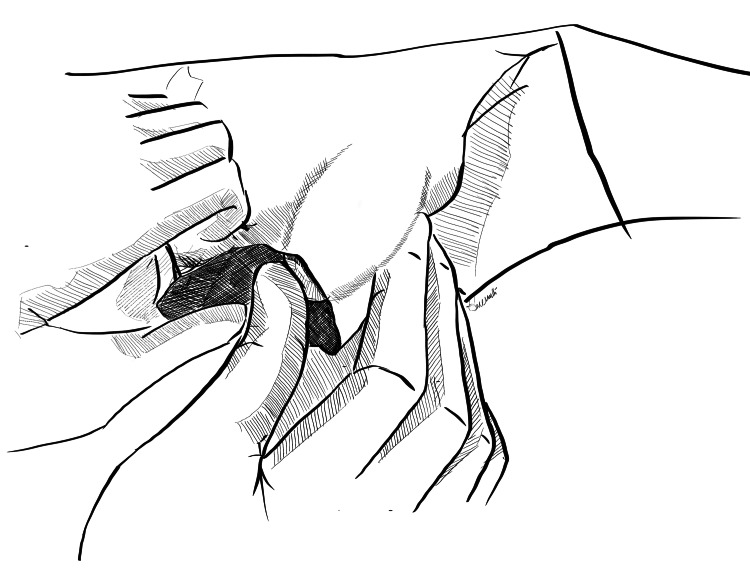
Placement of the testis in the left inguinal pouch The left testis is gently pushed manually into the inguinal pouch created. Original image created by Sreeshanth KS

Following creation of the inguinal pouch, the testes are washed again and placed in the inguinal pouch by pushing gently in the retrograde direction, making sure there is no torsion of the cord during placement (Figures 4, 5). After proper washing with saline, dressing of the residual scrotal sac is done.

Step 4: Repeat Debridement

The scrotal wound is dressed regularly. Various options, such as simple saline dressings and application of negative-pressure wound therapy (NPWT) device, are utilized to help in healthy granulation and healing of the scrotal wound. The patient is taken up for repeated surgical debridement when needed. During re-debridement, the testes are also brought down from the pouch and washed with saline. They are then placed back in the inguinal pouch.

Step 5: Closure of the Scrotum

Once healthy granulation tissue appears in the residual scrotal wound, the testes can be brought down to the scrotum. After mobilizing the residual scrotal skin, the scrotum is closed. However, in a few cases, skin grafting may be required to close the defect.

## Discussion

Fournier’s gangrene refers to a necrotizing fasciitis of the perineal region involving the genital, perianal, and other surrounding tissues [2]. It is a polymicrobial infection caused by both aerobes and anaerobes, which causes extensive tissue damage and subcutaneous vessel thrombosis and eventually gangrene. Though initial description by Jean Alfred Fournier in 1883 attributed this spreading scrotal infection to be idiopathic [3], further study into the etiology of this condition has revealed three main causes. These include urogenital causes such as perurethral catheterization, urethral stricture, vasectomy, prostate biopsies, epididymitis, and chronic urinary tract infection; anorectal causes such as hemorrhoid surgeries, colorectal malignancies, anal intercourse, or local trauma; and dermatological causes such as animal or human bites, burns, piercings, and injections. The commonly implicated organisms include aerobes such as *Escherichia coli*, *Staphylococcus*, and *Klebsiella*, and anaerobes such as *Bacteroides*. They are frequently seen in elderly males with comorbidities; however, young males, females, and children are not any exception to this disease [4]. Diabetes mellitus, chronic alcoholism, and obesity are some of the commonly associated comorbidities. The use of various scores such as Fournier’s gangrene severity index has been described to predict the mortality in these cases, based on the degree of derangement of physiology [5]. In various series, the reported mortality rate ranges from 3% to 67% [6].

The management of Fournier’s gangrene needs to be prompt and emergent. Immediate resuscitation with intravenous fluids to correct dehydration and shock, administration of a combination of broad-spectrum antibiotics such as third-generation cephalosporins, aminoglycosides, metronidazole, or clindamycin [7], and correction of acidosis and deranged blood sugars in case of diabetics form a pivotal role in the initial management. Urgent surgical debridement to remove the source of sepsis is the definitive treatment in such cases [8].

Following surgical debridement, the surgeon is faced with a problem of shameful exposure of the testes and inadequate tissue to cover the exposed gonads. Active infective process precludes the chance of primary closure of wound at the same sitting as debridement. Thus, a temporizing measure to preserve the gonads is warranted in these patients. Methods such as placement of the testes in thigh pouches have been described in existing literature [9].

**Figure 6:**
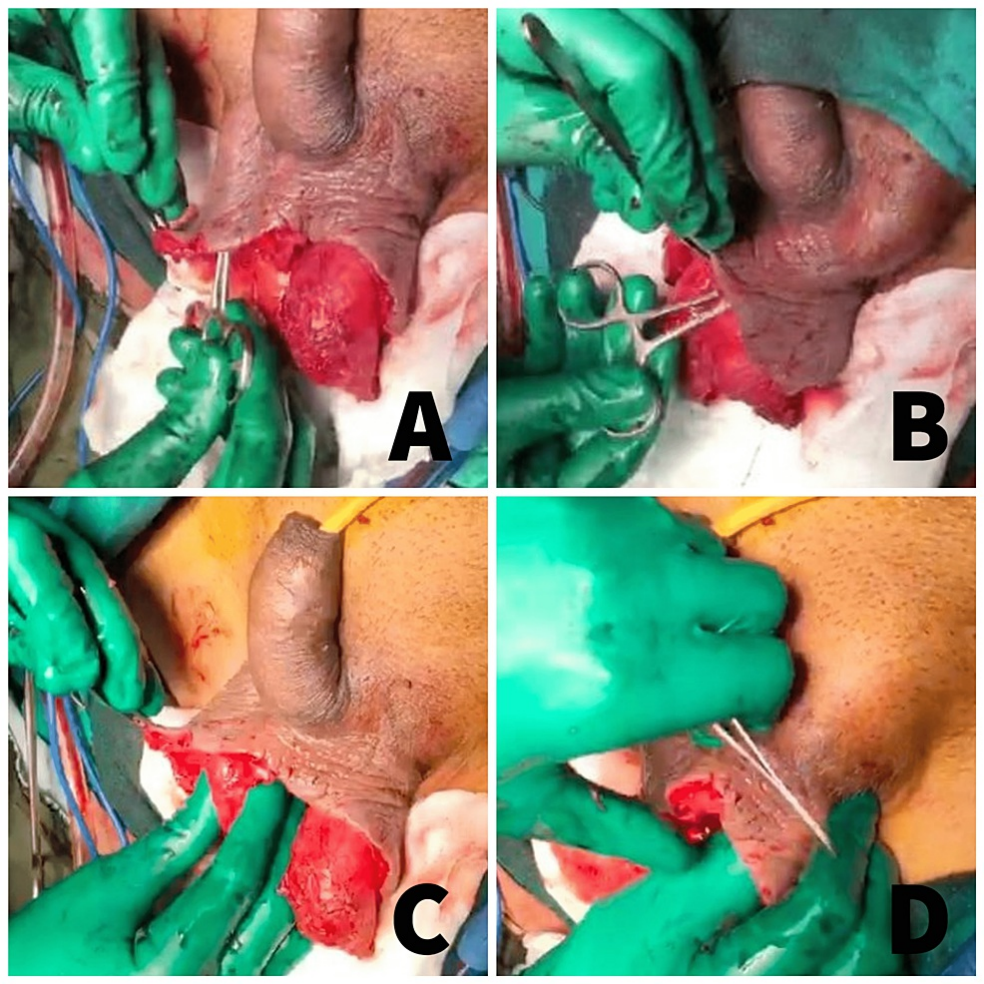
Clinical images of the technique of inguinal pouch creation and placement of the testes (A) Creation of the right inguinal pouch. (B) Creation of the left inguinal pouch. (C) Placement of the testis in the right inguinal pouch. (D) Placement of the testis in the left inguinal pouch.

Our study is unique in describing a novel technique of creation of the inguinal pouch for testicular preservation following debridement for Fournier’s gangrene. We applied this technique on 15 patients with Fournier’s gangrene. They were managed with appropriate antibiotics, immediate surgical debridement, and primary preservation of the testes in the inguinal pouch created (Figures 6A-6D).

**Figure 7:**
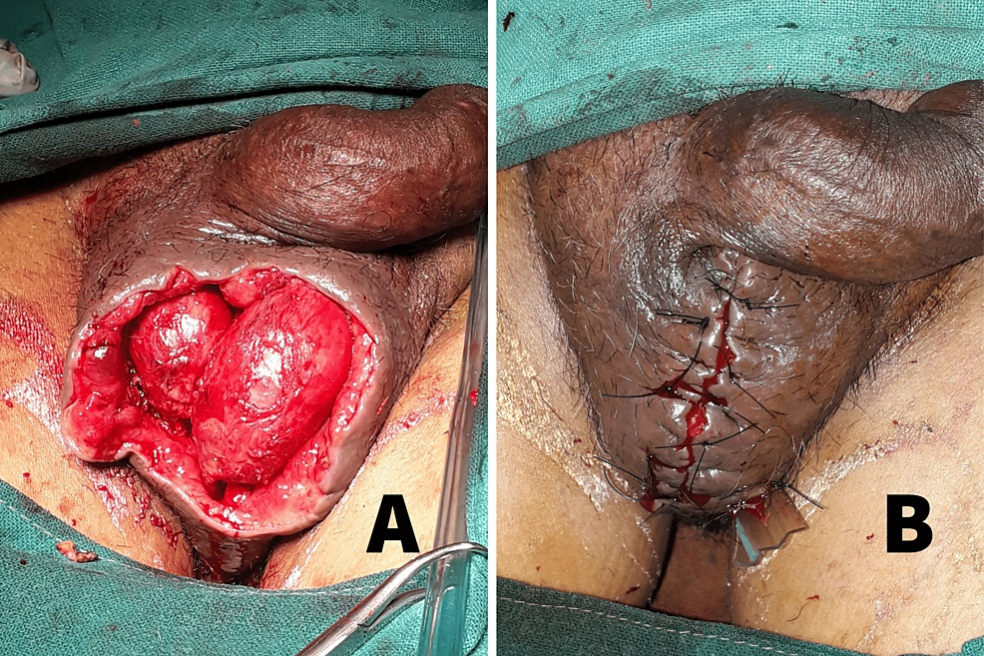
Clinical images of wound after re-debridement and primary closure of the scrotum (A) Scrotal wound with healthy granulation tissue following re-debridement. The testes have been brought down from the inguinal pouch for inspection. (B) Primary closure of the scrotum after bringing down the testes.

Following primary debridement, saline dressings were done regularly. Three patients also required NPWT. The testes could be brought down from the pouch, inspected, washed, and placed back during repeat debridements (Figure 7A). The number of re-debridements ranged from two to eight. The process was repeated until the wound appeared healthy with granulation tissue. In all our patients, the defect could be closed primarily after bringing the testes down by mobilizing the remaining scrotal tissue (Figure 7B). No complications such as local wound infection or testicular atrophy were documented following use of this technique. The use of an inguinal pouch to preserve the testes during the interim period was found to cause less physical and psychological trauma than when the testes were exposed. It was also quite simple to perform.

The choice of dressing following initial surgical procedure can vary from simple saline dressings to NPWT device. The use of NPWT for fastening the healing process is well-recognized and is attributed to improved angiogenesis [6]. After healthy granulation tissue develops, a plan for reconstruction of the scrotum is considered.

Various reconstruction techniques after replacement of the testes back to the scrotum have been used, such as delayed primary closure of the scrotal skin [10], and the use of local scrotal advancement flap, split-thickness skin graft, superomedial thigh flap, pudendal thigh flap, medial circumflex artery perforator flap, and gracilis flap [6]. Use of tissue expanders to mobilize the redundant scrotal skin locally has also been described [11]. Due to the laxity of the scrotal skin, a delayed primary closure is possible in most cases after repositing the testes to the scrotum, as observed in our study.

Overall, the advantages of this new technique include development of a relatively physiological position to preserve the testes before definitive reconstruction of the scrotum and the easy reproducibility of the technique. It also facilitates easy placement of the NPWT system, which might be problematic when the testes are exposed. The drawback includes the lack of randomized trials to understand the outcomes of this technique in comparison to the existing ones. Even though a theoretical risk of spread of infection to the unaffected inguinal region exists, none were observed in our patients. However, studies with a greater sample size would help to ascertain this outcome.

## Conclusions

Fournier’s gangrene is a surgical emergency that requires prompt resuscitation and debridement to control sepsis and prevent mortality. This technique of creation of an inguinal pouch for testicular preservation in case of Fournier’s gangrene is a novel and feasible approach to avoid shameful exposure of the gonads. An array of reconstruction techniques is available to shape the remaining scrotal skin to accommodate the testes. Adequate caution and prompt care, bearing in mind the psychological impact of this disease on the patient, completes the holistic approach to the management of Fournier’s gangrene.
